# Expert position paper on prolonged dual antiplatelet therapy in secondary prevention following myocardial infarction

**DOI:** 10.1007/s00508-016-1016-7

**Published:** 2016-06-09

**Authors:** Thomas W. Weiss, Josef Aichinger, Kurt Huber, Walter S. Speidl, Norbert Watzinger, Robert Zweiker, Hannes F. Alber

**Affiliations:** 3rd Medical Department of Cardiology and Intensive Care Medicine, Wilhelminenhospital, Vienna, Austria; Internal Department 2 – Cardiology, Angiology and Internal Intensive Medicine, Krankenhaus der Elisabethinen Linz, Linz, Austria; Clinical Department of Cardiology, University Clinic of General Medicine II, Medical University of Vienna, Vienna, Austria; Department of General Medicine, Landeskrankenhaus Feldbach, Feldbach-Fürstenfeld Hospital Group, Feldbach, Austria; Department of Cardiology, Medical University of Graz, Graz, Austria; University Clinic of General Medicine III at the Medical University of Innsbruck, Reha-Zentrum Münster, Tyrol and Karl Landsteiner Institute for Interdisciplinary Research at the Reha-Zentrum Münster, Münster/Tyrol, Austria

**Keywords:** P2Y_12_ inhibitor, Acute coronary syndrome, DAPT, Myocardial infarction, Ticagrelor

## Abstract

The protective effect of dual antiplatelet therapy (DAPT) following acute coronary syndrome is undisputed, but its duration is subject of debate. Several studies show that prolonged therapy provides a clinical benefit in patients following acute coronary syndrome. The aim of this position paper authored by Austrian experts is to outline the current evidence and provide an overview of recent studies. It is also intended to serve as a practical guide to identify those patients who may benefit from prolonged DAPT.

## Introduction

Platelet activation and aggregation with consecutive thrombus formation are key elements in the pathophysiology of ischaemic cardiovascular events [1]. Oral antiplatelet therapy is therefore a central measure in the management of acute coronary syndrome (ACS) and in the secondary prevention of cardiovascular events.

International guidelines on the management of non-ST elevation myocardial infarction (NSTEMI) and ST elevation myocardial infarction (STEMI) recommend immediate therapy with acetylsalicylic acid (ASA) and a P2Y_12_ inhibitor, both in patients who are undergoing conservative treatment and in patients undergoing interventional treatment. This dual antiplatelet therapy (DAPT) is to be continued for at least 12 months [2–6]. Tab. 1 provides an overview of the current guidelines.

**Table 1:** Current recommendations for dual antiplatelet therapy following an acute coronary syndrome

2015 ESC guidelines for the management of acute coronary syndromes in patients presenting without persistent ST-segment elevation [4]			2014 ESC/EACTS Guidelines on myocardial revascularization [3]		
Recommendations for platelet inhibition in non-ST-elevation acute coronary syndromes			Recommendations for antithrombotic treatment in patients with STEMI undergoing primary PCI		
	RC	RL		RC	RL
Aspirin is recommended for all patients without contraindications at an initial oral loading dosed of 150–300 mg (in aspirin-naive patients) and a maintenance dose of 75–100 mg/day long-term regardless of treatment strategy	I	A	ASA is recommended for all patients without contraindications at an initial oral loading dose of 150–300 mg (or 80–150 mg i. v.) and at a maintenance dose of 75–100 mg daily long-term regardless of treatment strategy	I	A
A P2Y_12_ inhibitor is recommended, in addition to aspirin, for 12 months unless there are contraindications such as excessive risk of bleeds	I	A	A P2Y_12_ inhibitor is recommended in addition to ASA and maintained over 12 months unless there are contraindications such as excessive risk of bleeding. Options are:	I	A
– Ticagrelor (180 mg loading dose, 90 mg twice daily) is recommended, in the absence of contraindications^a^, for all patients at moderate-to-high risk of ischaemic events (e. g. elevated cardiac troponins), regardless of initial treatment strategy and including those pretreated with clopidogrel (which should be discontinued when ticagrelor is started)	I	B	– Ticagrelor (180 mg loading dose, 90 mg twice daily) if no contraindication	I	B
– Prasugrel (60 mg loading dose, 10 mg daily dose) is recommended in patients who are proceeding to PCI if no contraindication^a^	I	B	– Prasugrel (60 mg loading dose, 10 mg daily dose) if no contraindication	I	B
– Clopidogrel (300–600 mg loading dose, 75 mg daily dose) is recommended for patients who cannot receive ticagrelor or prasugrel or who require oral anticoagulation	I	B	– Clopidogrel (600 mg loading dose, 75 mg daily dose), only when prasugrel or ticagrelor are not available or are contraindicated	I	B
It is not recommended to administer prasugrel in patients in whom coronary anatomy is not known	III	B	It is recommended to give P2Y_12_ inhibitors at the time of first medical contact	I	B
P2Y_12_ inhibitor administration for a shorter duration of 3–6 months after DES implantation may be considered in patients deemed at high bleeding risk	IIb	A			
P2Y_12_ inhibitor administration in addition to aspirin beyond 1 year may be considered after careful assessment of the ischaemic and bleeding risks of the patient	IIb	A			

*RC* Recommendation Class, *RL* Recommendation Level

^a^Contraindications for ticagrelor: previous intracranial haemorrhage or ongoing bleeds. Contraindications for prasugrel: previous intracranial haemorrhage, previous ischaemic stroke or transient ischaemic attack or ongoing bleeds; prasugrel is generally not recommended for patients ≥ 75 years of age or with a bodyweight < 60 kg

**Fig. 1:**
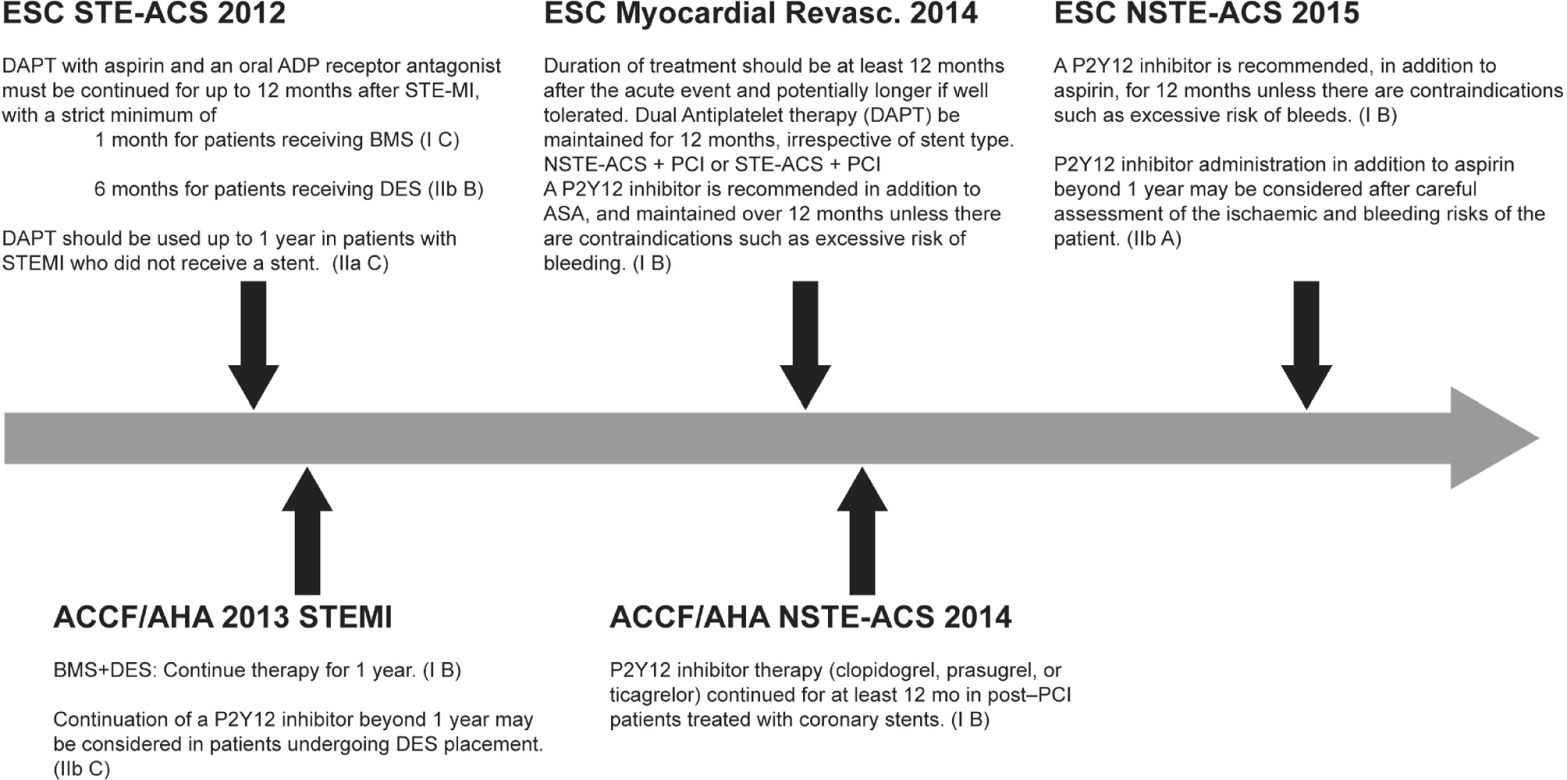
Recent changes in recommendations of prolonged dual antiplatelet therapy in international guidelines. *ESC* European Society of Cardiology, *STE-ACS* ST-Segment Elevation Acute Coronary Syndrome, *ACS* Acute Coronary Syndrome, *NSTE-ACS* Non-ST-Segment Elevation Acute Coronary Syndrome, *DAPT* Dual Antiplatelet Therapy, *BMS* Bare Metal Stent, *DES* Drug Eluting Stent, *STEMI* ST-Segment Elevation Myocardial Infarction, *PCI* Percutaneous Coronary Intervention, *ASA* Acetylic Salicylic Acid, *ACCF* American College of Cardiology Foundation, *AHA* American Heart Association

In certain situations, it is recommended to consider the extended administration of DAPT beyond the 12-month period (Fig. 1; [4–6]). According to the *2013* American College of Cardiology Foundation/American Heart Association* (ACCF/AHA) Guideline for the Management of STEMI*, therapy with a P2Y_12_ inhibitor beyond a year may be considered in high-risk patients who receive a drug-eluting stent (DES; IIb C; [5]). The *2014 ACCF/AHA Guideline for the Management of Patients with Non-ST Elevation Acute Coronary Syndromes* recommends P2Y_12_ inhibitor therapy for at least 12 months in patients after a percutaneous coronary intervention (PCI) with stent implantation (I B) [6]. The most recent guideline issued by the European Society of Cardiology (ESC) for non-ST elevation ACS state that a P2Y_12_ inhibitor therapy beyond one year may be considered after carefully taking into consideration the patient’s ischaemic and haemorrhagic risk (IIb A) [4].

## Rationale for prolonged DAPT

**Fig. 2:**
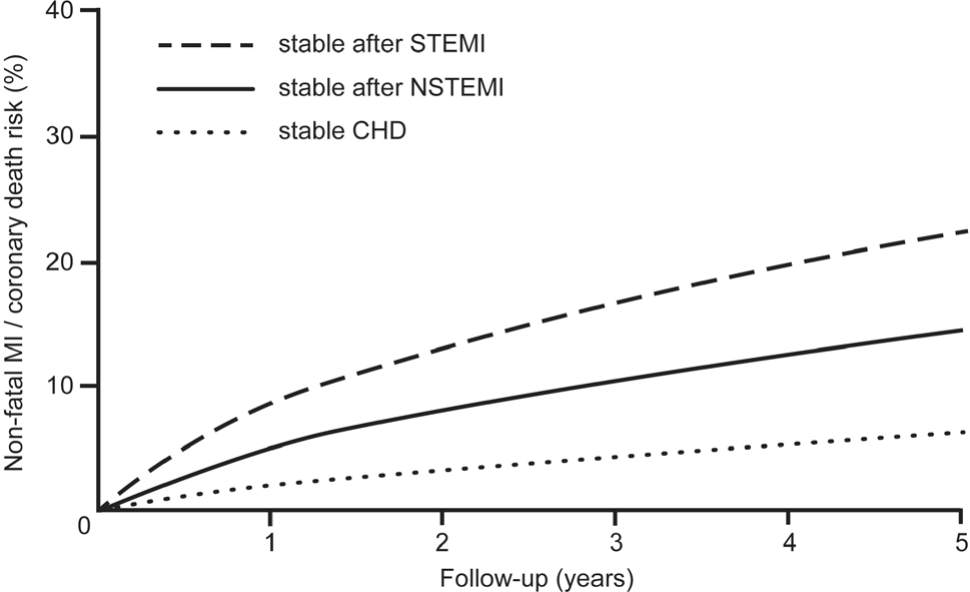
Kaplan–Meier risk (nonfatal MI or coronary death) for stable angina patients (*n* = 45,645), STEMI (*n* = 4,700) and NSTEMI (*n* = 6,818). For ACS patients, follow-up started 6 months after index event. Mean follow-up time was 4.4 years. Adapted from [9] *CHD* coronary heart disease, *MI* myocardial infarction, *NSTEMI* non-ST-elevation myocardial infarction, *STEMI* ST-elevation myocardial infarction

The rationale for prolonged DAPT is the fact that the ischaemic risk of patients following myocardial infarction remains high beyond the first year [7, 8]. This is shown, for example, by Swedish record data from more than 100,000 patients who were hospitalised with myocardial infarction. The risk of these patients suffering a severe cardiovascular event (nonfatal myocardial infarction, nonfatal stroke or cardiovascular death) in the first year after the acute event was ~ 18 %. Event-free patients in the first year still had an approximate 20 % risk of an event in the following three years. The probability of suffering such an event was linked to the number of cardiovascular risk factors. Older age, stroke, diabetes, heart failure and no index revascularisation were independently associated with an increased risk of ischaemic events or mortality [8]. The prolonged increased risk in stable patients following an ACS in comparison with patients with stable coronary artery disease without ACS was also demonstrated in British record data, in which the 5‑year risk of infarction or sudden cardiac death was approximately double in patients with STEMI, and almost triple in patients with NSTEMI (Fig. 2; [9]).

The long-term increased risk of recurrence is based on the fact that the treatment of the acute event does not constitute causal therapy of atherosclerosis itself, which is a progressive, systemic disease that is not limited to a culprit lesion. It has been shown that only half of recurrent ischaemic cardiac events in patients who have undergone a successful percutaneous coronary intervention (PCI) in relation to ACS are caused by the culprit lesion, while the other half are caused by non-culprit or de novo lesions [10]. (For further literature on the subject of plaque vulnerability, see [11]). Therefore, intensive secondary prevention with rigorous control of all individual risk factors (blood pressure, blood sugar, lipids) is crucial for the patient’s prognosis.

## Risk and benefit of prolonged DAPT

Prolonged DAPT not only has the potential to reduce the stent thrombosis risk, but is also associated with a reduced risk of recurrent infarctions [12–14].

Although the Clopidogrel for High Atherothrombotic Risk and Ischemic Stabilization, Management, and Avoidance (CHARISMA) study on prolonged DAPT with clopidogrel/ASA over 28 months did not show any benefit of prolonged DAPT with regard to cardiovascular risk for the total patient cohort [15], a retrospective subgroup analysis revealed that the prolonged DAPT significantly reduced the risk of major cardiovascular events in the secondary prevention setting after myocardial infarction [12].

In the Dual Antiplatelet Therapy (DAPT) study, prolonged DAPT over 30 months was compared with DAPT over 12 months. This study included patients with stable coronary artery disease or acute coronary syndrome, provided they had received at least one stent. A subgroup analysis showed that in patients both with and without myocardial infarction, the extended DAPT lowered the risk of stent thrombosis. In patients with myocardial infarction, the extended DAPT also reduced the risk of major cardiovascular events (cardiovascular death, myocardial infarction, stroke) [13].

In addition to these studies, the Platelet Inhibition and Patient Outcomes (PLATO) study points towards a benefit of prolonged DAPT. More intensive platelet inhibition with ticagrelor 90 mg twice daily reduced the risk of cardiovascular morbidity and mortality in comparison with clopidogrel 75 mg once daily not only in the early phase after the acute event, but also continuously and incrementally in the more stable phase until the end of follow-up up to 12 months [16].

## Extended DAPT with ticagrelor 60 mg twice daily (PEGASUS-TIMI-54 study)

The first prospective study to examine the effect of prolonged DAPT in post-MI patients was the Prevention of Cardiovascular Events in Patients with Prior Heart Attack Using Ticagrelor Compared to Placebo on a Background of Aspirin-Thrombolysis in Myocardial Infarction 54 (PEGASUS-TIMI-54) study published in 2015 [17].

### Study population, study medication, endpoints

This double-blind, placebo-controlled study involved 21,162 high-risk patients with a history of myocardial infarction and at least one other cardiovascular risk factor (diabetes mellitus, re-infarction, aged ≥ 65 years, multivessel coronary artery disease, chronic non-end-stage renal dysfunction). These patients were included in the study one to three years after the acute event [17, 18]. In the first year after the myocardial infarction, the DAPT treatment was given independently of the PEGASUS-TIMI-54 trial. As the patients were included between October 2010 and April 2013 and the qualifying myocardial infarction must have happened one to three years before that time, the majority of the patients received clopidogrel in the first year following their ACS. At that time, prasugrel had only recently been launched on the market and ticagrelor only became available for general clinical use during the course of the PEGASUS trial.

Key exclusion criteria included patients with a history of ischaemic stroke, recent haemorrhage or oral anticoagulant therapy.

The patients were randomised in a ratio of 1:1:1 to receive ticagrelor 90 mg twice daily, ticagrelor 60 mg twice daily or placebo. Patients received ASA at a low dosage (maintenance dose ≤ 150 mg) and were followed up for a period of 33 months. The primary efficacy endpoint was the combination of cardiovascular death, myocardial infarction or stroke. The primary safety endpoint was major haemorrhage as classified by the Thrombolysis in Myocardial Infarction (TIMI) criteria (fatal haemorrhage, intracranial haemorrhage, a reduction in haemoglobin ≥ 5 g/dl or a reduction in haematocrit ≥ 15 %) [19].

### Results

#### Efficacy data

**Fig. 3:**
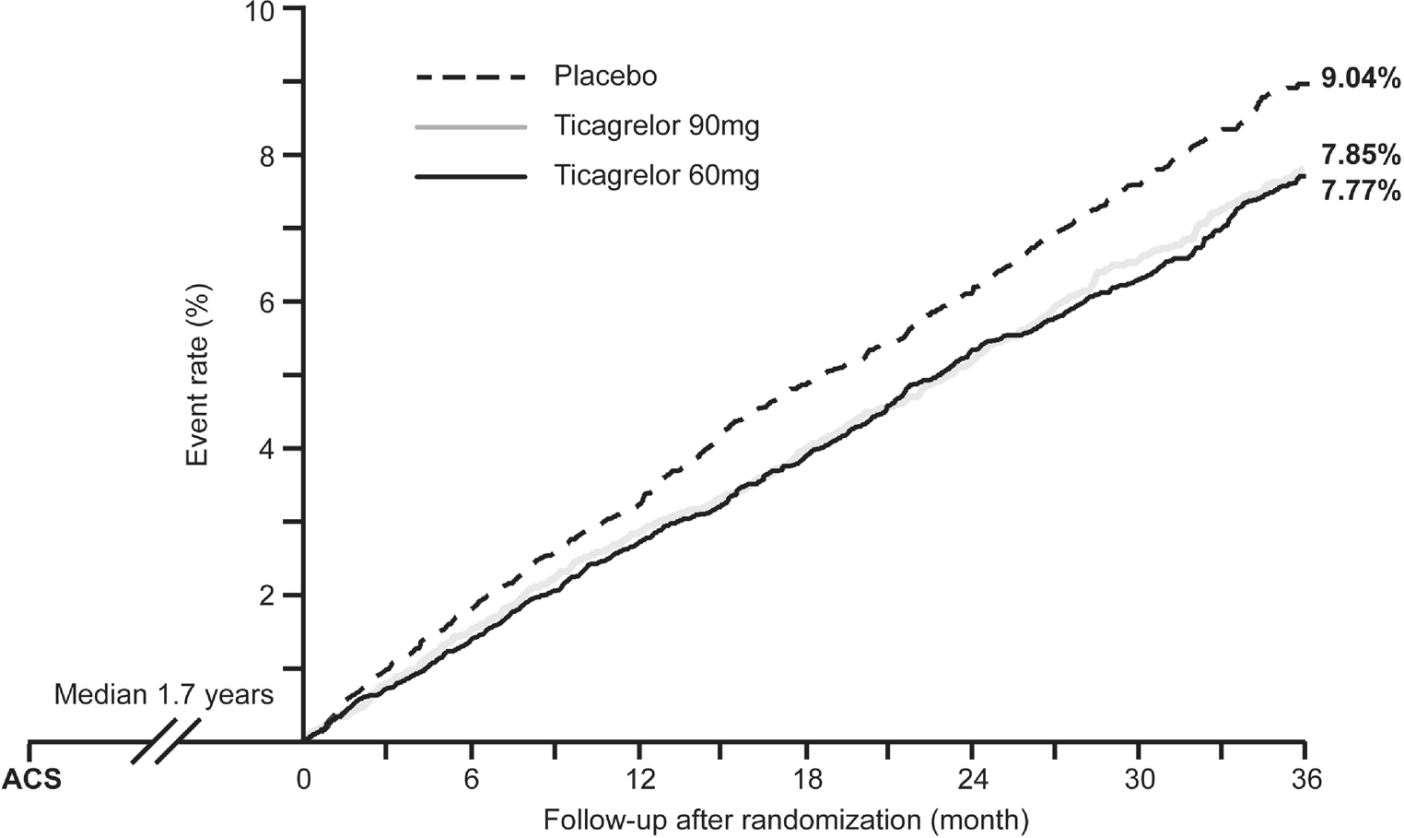
Patients were randomized 1–3 years (median 1.7 years) after index myocardial infarction (*MI*). Kaplan–Meier rates of cardiovascular death, myocardial infarction, or stroke over 3 years were 9.04 % in the placebo group and 7.85 % in the group that received 90 mg ticagrelor twice daily (*BI* *D*; vs placebo HR 0.85; 95 % CI 0.75–0.96; *p* = 0.008) and 7.77 % in the group that received 60 mg ticagrelor twice daily (vs placebo HR 0.84; 95 % CI 0.74–0.95; *p* = 0.004). Adapted from [17]. *ACS* acute coronary syndrome

At both dosages, ticagrelor significantly reduced the risk of the primary efficacy endpoint in comparison with placebo (Kaplan–Meier rates at year 3 – ticagrelor 90 mg twice daily: 7.85 %; ticagrelor 60 mg twice daily: 7.77 %; placebo: 9.04 %; Fig. 3). The hazard ratio (HR) for ticagrelor 90 mg twice daily versus placebo was 0.85 (95 % confidence interval [CI]: 0.75–0.96; *p* = 0.008) and the HR for ticagrelor 60 mg twice daily versus placebo was 0.84 (95 % CI: 0.74–0.95; *p* = 0.004).

The comparability of the efficacy and safety of ticagrelor 90 mg twice daily and ticagrelor 60 mg twice daily have been reflected in the approval of ticagrelor 60 mg twice daily for prolonged DAPT by the European Medicines Agency (EMA) on 19 February 2016. Therefore, only the data for the ticagrelor dosage that is clinically relevant for prolonged DAPT, 60 mg twice daily, will be quoted in the following sections.

**Fig. 4:**
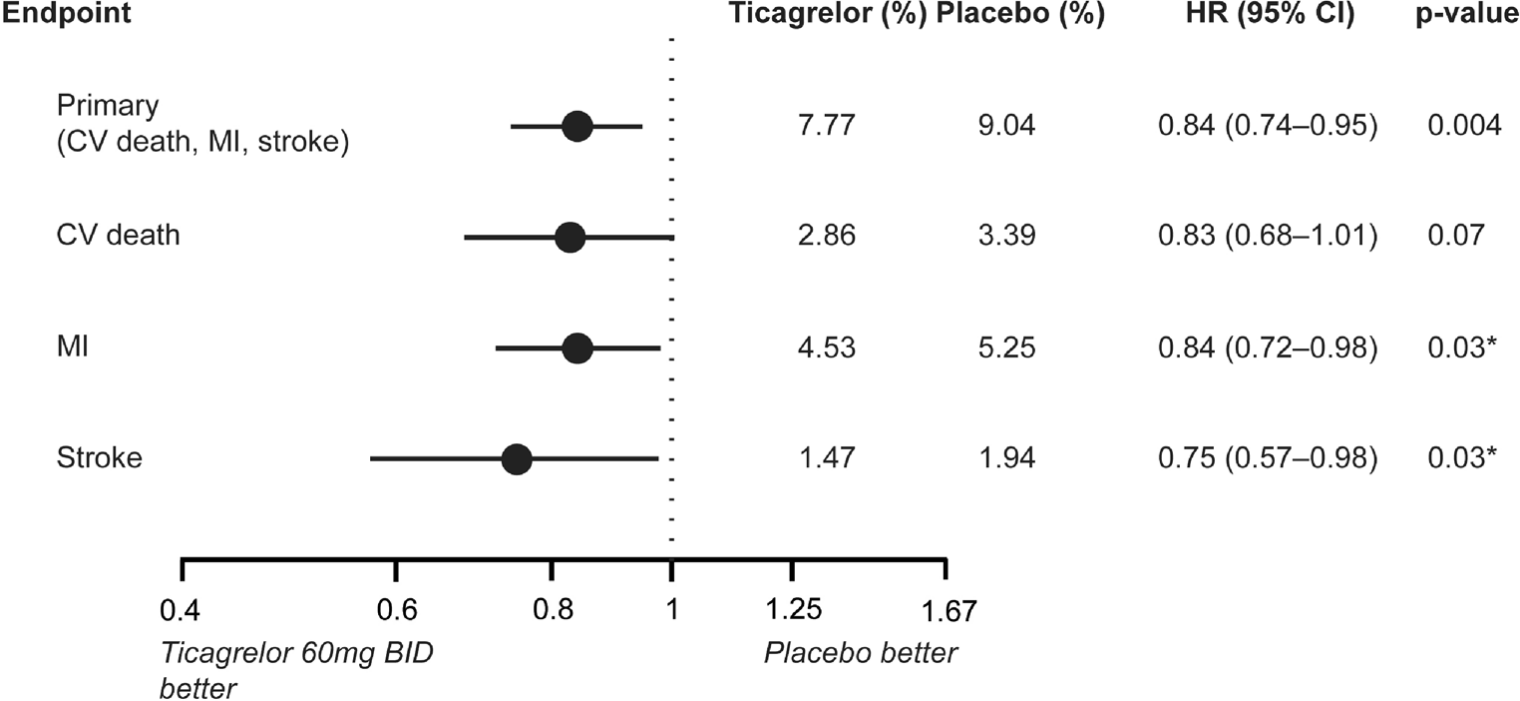
Effects of ticagrelor 60 mg twice daiy + ASA versus placebo + ASA on the combined primary efficacy endpoint (CV death, MI, stroke) and individual components. After [17]. *CV* cardiovascular, *MI* myocardial infarction, *ASA* acetylsalicylic acid, *CI* confidence interval

The exploratory analysis showed that ticagrelor 60 mg twice daily versus placebo resulted in a significant reduction in the rate of myocardial infarction, a reduced rate of stroke and comparable cardiovascular mortality. Fig. 4 provides an overview of the evaluation of the individual efficacy endpoints of the primary combined endpoint.

For every 10,000 patients who start prolonged DAPT with ticagrelor 60 mg twice daily (intention-to-treat analysis), 42 primary endpoint events can be prevented every year.

#### Safety data

**Fig. 5:**
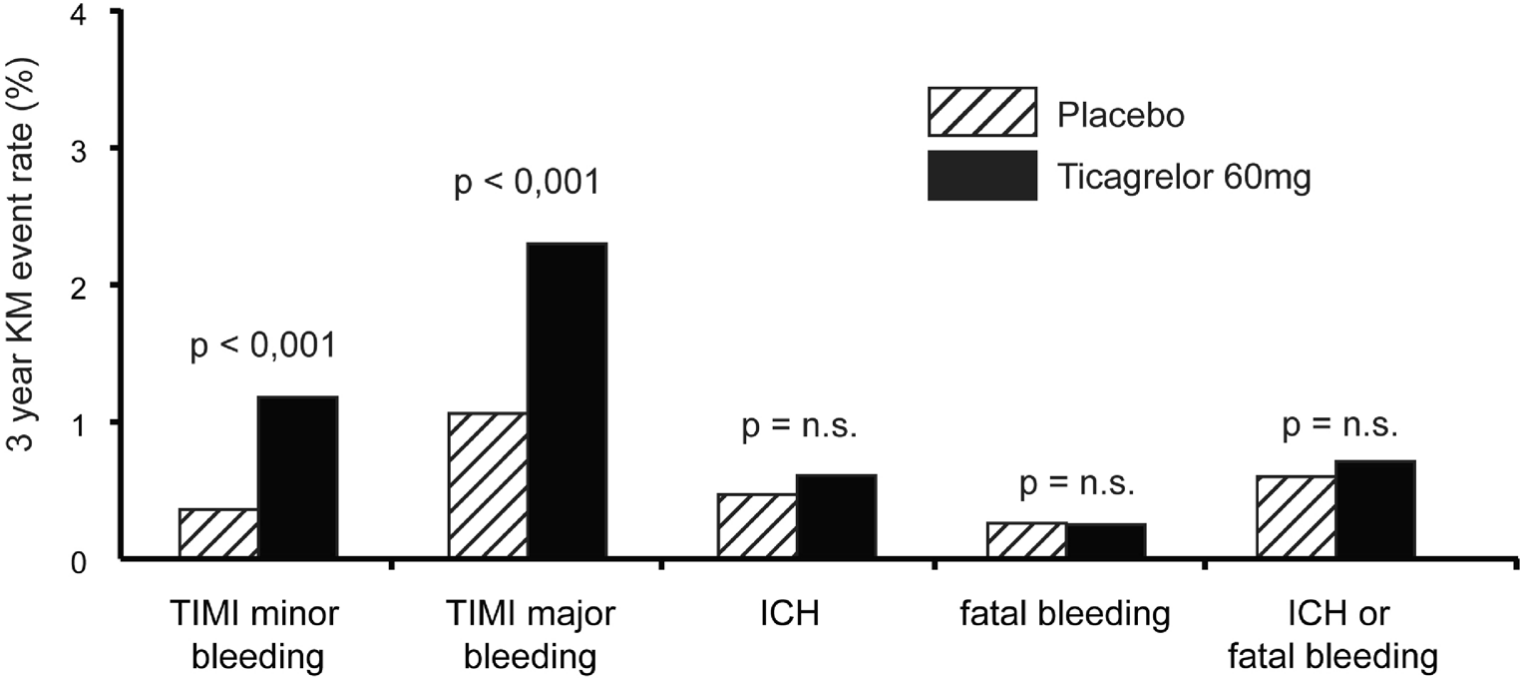
Cumulative event rates of TIMI major bleeding at 3 years were 1.06 % in the placebo group, 2.6 % in the ticagrelor 90 mg twice daily group and 2.3 % in the ticagrelor 60 mg twice daily group. The 3 year Kaplan–Meier (*KM*) rates for fatal bleeding or intracranial haemorrhage (*ICH*) were 0.6, 0.63 and 0.71 %, respectively. *n.s.* not significant, *TIMI* Thrombolysis in Myocardial Infarction

Over a period of three years, the frequency of TIMI major bleedings was significantly higher with ticagrelor 60 mg twice daily than with placebo (2.30 % vs. 1.06 %; *p* = 0.001), while the rates of intracranial or fatal haemorrhage were comparable (0.71 % vs. 0.60 %). Fig. 5 shows the cumulative bleeding event rates at 3 years.

Over a three-year period, dyspnoea was more frequent with ticagrelor 60 mg twice daily in comparison with placebo (15.84 % vs. 6.38 %; *p* < 0.001). This side effect was mostly mild or moderate and in many cases only temporary. Hence, the discontinuation rates due to dyspnoea were much lower at only 4.55 % in the ticagrelor 60 mg arm (placebo: 0.79 %; *p* < 0.001). Discontinuation of therapy due to dyspnoea occurred soon after initiation of therapy.

Renal events and bradyarrhythmias occurred in the treatment groups at similar frequencies. Severe episodes of gout were documented more frequently with ticagrelor than with placebo.

#### Further analyses

**Fig. 6:**
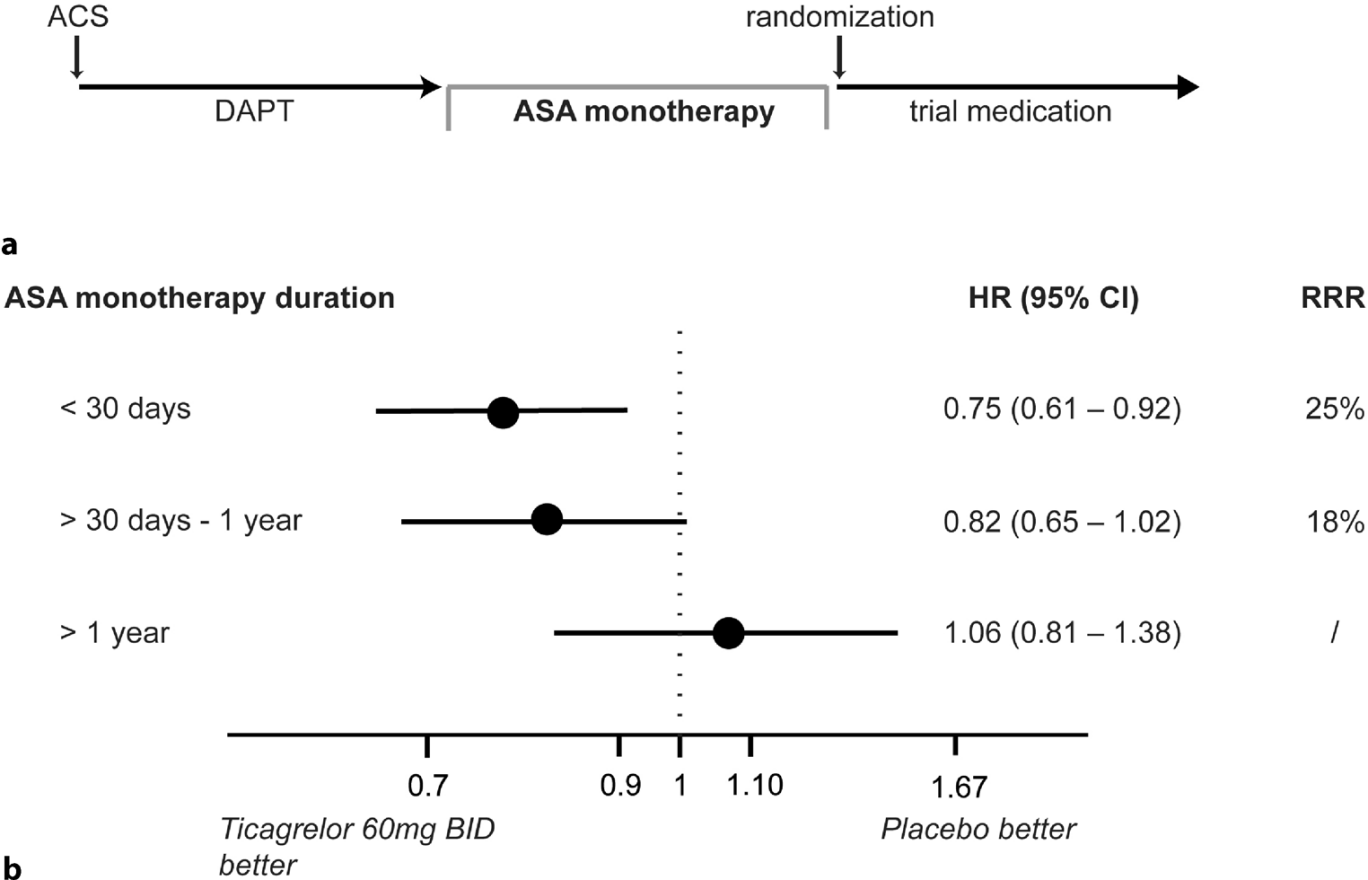
**a** Timeline of patients enrolled in trial. After the qualifying ACS patients were treated with DAPT independent of the study. After DAPT withdrawal patients were treated with ASS monotherapy until randomization to ticagrelor or placebo. **b** Analysis of 3‑year rate of efficacy endpoint (CV death, stroke, MI) according to time from last P2Y_12_ inhibitor to randomization (= ASA monotherapy phase). days: ≤ 30, > 30–360, > 360. Data is shown for ticagrelor 60 mg twice daily vs placebo. Adapted from [20]. *ASA* acetylsalicylic acid, *ACS* acute coronary syndrome, *DAPT* dual antiplatelet therapy, *HR* hazard ratio, *RRR* relative risk reduction

- Patients who started treatment with ticagrelor 60 mg twice daily within a short time (≤ 30 days of ASA monotherapy) after the end of the initial DAPT received a greater benefit than patients in whom DAPT was stopped for a longer period of time (Fig. 6; [20]).
- The rate of haemorrhage resulting in irreversible damage or death was < 1 % in all groups over the three-year period, without any statistically significant difference between ticagrelor and the placebo group.
- The analysis of the primary efficacy endpoint in combination with the primary safety endpoint of TIMI major bleedings showed no significant difference between ticagrelor and placebo. However, in terms of the combined benefit/safety analysis of ischaemic endpoints and bleeding events with *irreversible* damage (i. e. intracranial and fatal haemorrhage), prolonged DAPT with ticagrelor 60 mg twice daily demonstrated a benefit in comparison with placebo [21].
- Already in the first 12 months after an ACS, ticagrelor proved to be particularly beneficial in patients with stage III kidney disease as shown in the PLATO study [16]. This tendency can also be observed in PEGASUS [22].

## Recommendation for the use of prolonged DAPT with ticagrelor 60 mg twice daily/ASA 100 mg in patients following myocardial infarction

**Fig. 7:**
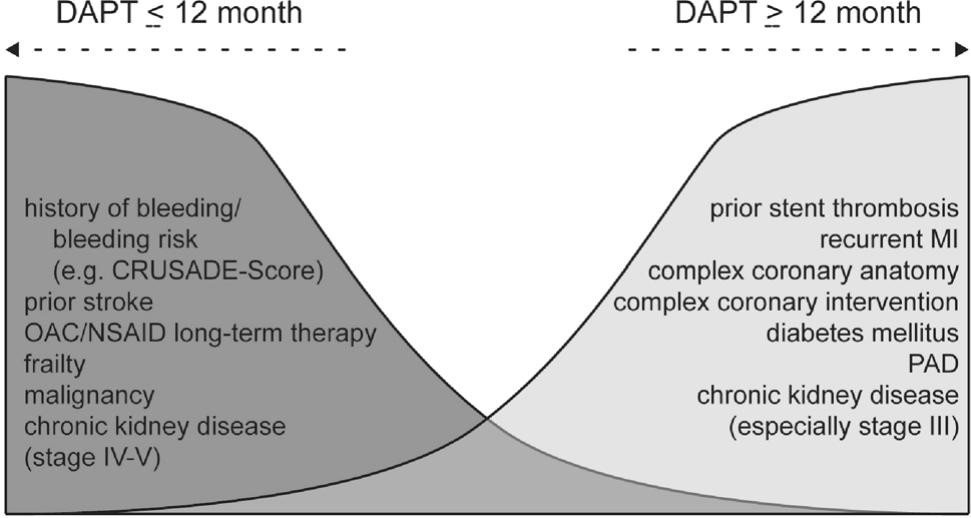
Careful evaluation of the cardiovascular risk factors and bleeding risk factors should determine the recommendation of DAPT duration at the time of discharge from the hospital after MI. *OAC* oral anticoagulation, *PAD* peripheral artery disease

In addition to optimum control of cardiovascular risk factors (lipids, blood sugar and blood pressure, smoking cessation, weight control), the following procedure can be recommended for prolonged DAPT:

- *Patient selection:* The prerequisite for the indication of prolonged DAPT is the individual evaluation of the ischaemic and bleeding risk. Prolonged DAPT is recommended accordingly in patients demonstrating one of the following characteristics: Stent thrombosis, re-infarction, complex coronary anatomy, complex intervention, overt diabetes mellitus, peripheral arterial disease (PAD), non-end stage chronic kidney disease (especially stage III) (see Fig. 7). Regardless of cardiovascular risk, the following patients should rather not receive prolonged DAPT: Patients with a history of haemorrhage or at high risk of haemorrhage (e. g. a CRUSADE score > 40) [23], a history of TIA or stroke, patients on oral anticoagulant therapy or under continuous treatment with nonsteroidal anti-inflammatory drugs (NSAIDs), frail patients, patients with malignancies and patients with stage IV–V chronic renal disease.
- *Indication:*
  1. *In an acute event by the treating interventional cardiologist:* The main indication for prolonged DAPT with ticagrelor 60 mg twice daily should be determined at the time of the acute event, documented in the discharge letter and explanatory argument. This is an easy timepoint at which the complexity of the intervention and of the coronary anatomy can be assessed.
  2. *By the rehabilitation physician:* Inpatient/outpatient rehabilitation offers a good opportunity to inform patients about the value of prolonged DAPT if the decision was not made at the acute hospital, and the initial tolerability of DAPT can be assessed under medical supervision.
  3. *Within one year from the acute event, e. g. by a resident specialist for internal medicine:* Before the end of the standard 12-month DAPT, the initial indication for prolonged DAPT with ticagrelor 60 mg twice daily should be reassessed on the basis of the tolerability of the now almost one-year DAPT (no clinically significant haemorrhage) and the persistently high ischaemic risk. If indicated, the prolonged DAPT should be continuously prescribed.
- *Notes for primary care providers:*
  - When using ticagrelor in the first year after myocardial infarction, the recommended dose is 90 mg twice daily [2–6]. The corresponding dose for prasugrel is 10 mg once daily (except in elderly and low weight patients in whom a dose reduction to 5 mg is recommended) and for clopidogrel 75 mg once daily in this first year. Prolonged DAPT with ticagrelor has been approved at a dose of 60 mg twice daily.
  - *At 12 months following ACS*, ticagrelor 90 mg twice daily, prasugrel 10 mg once daily or clopidogrel 75 mg once daily can be switched directly to ticagrelor 60 mg twice daily (with no loading dose). As far as possible, DAPT with ticagrelor 60 mg twice daily should be continued without interruption following the 12-month DAPT after ACS.
  - Ticagrelor 60 mg twice daily is permitted as part of prolonged DAPT provided that therapy is initiated within two years after the acute event or within one year after the end of a preceding course of DAPT.
  - Ticagrelor 60 mg twice daily is permitted for long-term therapy; study data over a period of three years is available.
